# Net2Align: An Algorithm For Pairwise Global Alignment of Biological Networks

**DOI:** 10.6026/97320630012408

**Published:** 2016-12-04

**Authors:** Priyanka Narad, Ankur Chaurasia, Gulshan Wadhwab, K. C. Upadhyayaa

**Affiliations:** 1 Amity Institute of Biotechnology, Amity University Uttar Pradesh, U.P., India;; 2 Joint Director, Department of Biotechnology, CGO Complex, New Delhi, India;

**Keywords:** Algorithm, Pairwise Global Alignment, Biological Networks

## Abstract

The amount of data on molecular interactions is growing at an enormous pace, whereas the progress of methods for analysing this data is still lacking behind. Particularly, in the area of comparative analysis of biological networks, where one wishes to explore the similarity between two biological networks, this holds a potential problem. In consideration that the functionality primarily runs at the network level, it advocates the need for robust comparison methods. In this paper, we describe Net2Align, an algorithm for pairwise global alignment that can perform node-to-node correspondences as well as edge-to-edge correspondences into consideration. The uniqueness of our algorithm is in the fact that it is also able to detect the type of interaction, which is essential in case of directed graphs. The existing algorithm is only able to identify the common nodes but not the common edges. Another striking feature of the algorithm is that it is able to remove duplicate entries in case of variable datasets being aligned. This is achieved through creation of a local database which helps exclude duplicate links. In a pervasive computational study on gene regulatory network, we establish that our algorithm surpasses its counterparts in its results. Net2Align has been implemented in Java 7 and the source code is available as supplementary files.

## Background

During the last decade, an enormous growth has been seen in terms of biological network data. This data include information on Gene Regulatory Network (GRN), Protein- Protein Interaction Network (PPI) and Metabolic Pathway [1, 2,3, 4]. As the number of biological networks is becoming available for analysis, it has become imperative to find out approaches for comparison of networks. These comparisons help us extrapolate the information to species-specific evolution and divergence amongst the various pathways. One of the most common approaches in this context is the network alignment. It defines finding similarities between the topology of the two (pairwise) or more (multiple) networks. This is useful in principle since we can transfer the information of one node and its corresponding edge to the same node in different network if they are aligning [5].

Previously, most of the methods used have been on local alignment [6]. These find local regions of similarity, detecting conserved clusters or patterns. Though, useful to identify conserved modules, local alignment can be ambivalent. However, global alignment provides a unique approach where each node in one network is mapped across each node in another network. In particular, ISORANK [7] aimed to provide maximum overall match between two networks. It used a heuristic by assigning a score to the aligning pair where 2 nodes are considered a match if their neighbors also match. An advanced version of this algorithm was ISORANKN [8], which relies on node specific ranking. Also, NATALIEQ [9] was developed which is a web server for topology-based alignment of a PPI with a selected target network from their database.

Nevertheless, all the approaches employed so far works implicitly on the topology of the network and a priori information is needed pertaining to sequence similarities of the genes/proteins in the network. Though, this works in case of an undirected network such as PPI, but in case of other network representations such as GRNs and Metabolic pathway, all edges are not equal. The network in this case is a directed one, where edges indicate different types of interactions. Alignment of this type of representation such as activation, inhibition or auto-regulation is relatively difficult or not possible using the existing algorithm. Our method does not rely on any sequence information only; it can align any type of biological network-directed/undirected. In the next section, we discuss the implementation, input requirements and case study where we align two biological networks representing a complex process of pluripotency in human and its most closely related organism mouse.

## Methodology

### Algorithm

**Figure 1:**
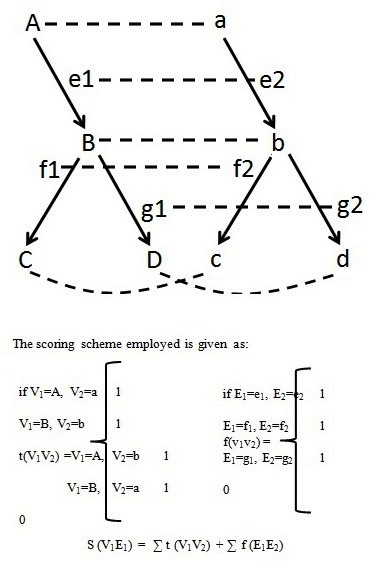
An illustration of network alignment is shown

Given two simple directed networks, N1 and N2, we have N1= (V1 E1) and N2= (V2 E2). An alignment is performed between N1 and N2. We consider that every node in N1 has a corresponding node in N2 as depicted below using (Figure 1) For any two biological networks represented by N1 and N2 with above-mentioned notations, scoring is first done for all the nodes in the two networks. For each node a comparison is drawn to the corresponding node in the second network, and a score is assigned for every column match. Once both the columns are matched for the entire dataset, the total score t (V1V2) is calculated. The algorithm takes into account the interchangeability of the positions of Node1 and Node2 and does not produce duplicated results if the two columns are same. The second level of scoring is performed for the edges. Each edge is then matched across the corresponding edge in the second network. A score is assigned for each corresponding edge that matches its counterpart, and a total score f (E1E2) is calculated. The final score is then calculated by the denoted by S (V1E1), which is the summation of the total number of nodes and edges that match. This is highly beneficial in case of GRNs, where the type of reaction gives us biological insights of the process under consideration.

### Implementation

Net2Align has been designed using JDK 8.1 which is a complete java development package comprising several APIs. To develop this algorithm, we have used Netbeans 8.1 IDE. NetBeans IDE is an official IDE for Java 8. It comprises with editors, code analyzers, and converters, using which we can write and develop our programs quickly. Netbeans IDE is also equipped with several inbuilt tools, which are capable of identifying and fixing the common problems in java code. This helped us to develop a bug free algorithm. The basic input required is the network data in the form of a database with .accdb extension. The input page consists of two push buttons, clicking on each will open a dialog box asking for the path of network database to be uploaded server side (Figure 2A, 2B, 2C). After choosing both network databases there is a push button to upload these files. The databases will be uploaded and matched server side and the final result is evaluated. The output is in the form of servlet response. This would be sent back to the user who has generated the request. The user as a web page containing result in a tabulated form can view output. The user can view this web page by using web browser like Chrome, Mozilla etc.

## Discussion

**Figure 3:**
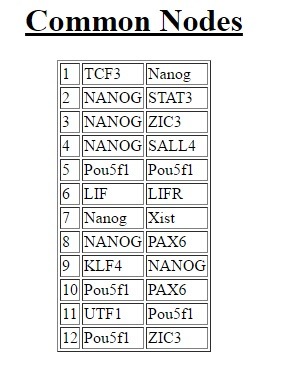
Common Nodes. Net2Align highlights the common nodes between the two networks treating the network as an undirected graph generating a two-column output.

**Figure 4:**
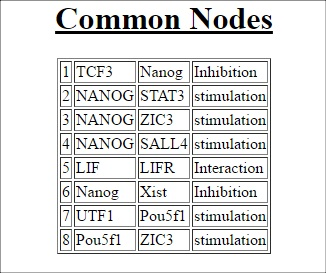
Common Nodes and Edges. Net2Align highlights the common nodes and edges between the two networks treating the network as a directed graph generating a three-column output.

To demonstrate the performance of our algorithm, we take two biological networks based on human and mouse pluripotency. Human pluripotency network consists of 122 nodes and 166 edges (unpublished data), whereas the mouse pluripotency network consists of 274 nodes and 574 edges [10]. The comparison was aimed at reproducing a conserved/divergent node based comparison between the two species. Net2Align was used to perform a pair-wise global alignment between these two biological networks representing similar complex phenomenon. Net2Align highlighted common nodes and edges between the two networks (Figure 3). The key reason for conducting this study was to enlighten the research community that one must be cautious in the extrapolation of data generated by current as well as previous studies utilizing mouse ES cell lines to that of human ES cell lines, as such acts may result in false study inferences. On the experimental level, a number of studies have been undertaken where comparisons have been made between the two species [11]. Our observations also concluded that the common links between human and mouse pluripotency network include the shutdown around NANOG and POU5F1. The conservation between the two species can be inferred through the links around these two transcription factors. Both the network considered were directed networks consisting of the third column showing the type of interaction. We also treated them as undirected by removing the third column and only matching the nodes that are common between the two networks. We observed that the number of common nodes increase in number when we consider them as undirected (Figure 4).

## Conclusion

In this paper, we presented Net2Align, an algorithm for computation of pairwise global alignment of all types of biological networks particularly useful for GRNs. To the best of our knowledge, it is the first algorithm for computation of edge-based comparison of biological network providing an exact match between the two aligned networks. In future, we intend to offer a user-friendly interface and an easy 2D visualization of global alignment between the networks. Moreover, alignments can be further investigated, by attaching GO terms to the proteins of aligning networks. We believe that Net2Align can be a valuable tool to better understand the functioning of biological processes and species specificity. It can help to transfer functional annotations from one species to another, to predict new complexes or to help in establishing the function of unknown proteins within the cells with the help of its known matched partner.

## Figures and Tables

**Figure 2A:**
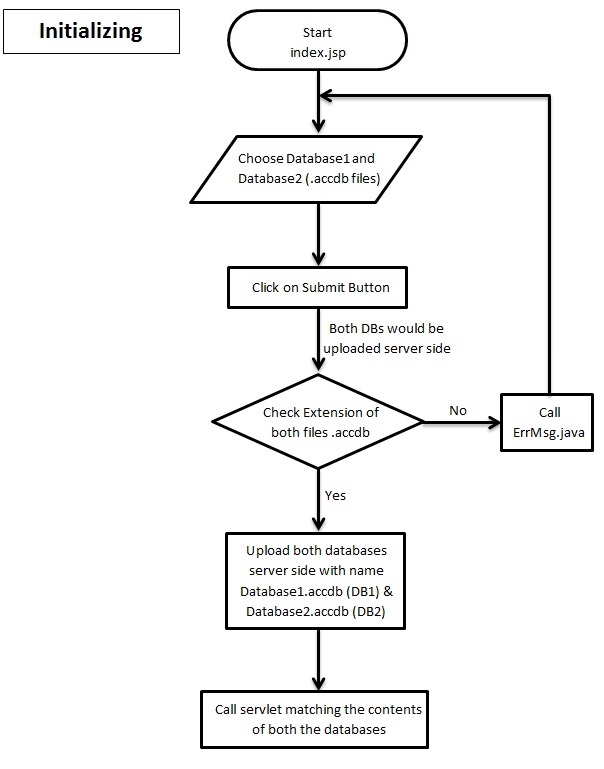
Flowchart describing uploading databases server side

**Figure 2B:**
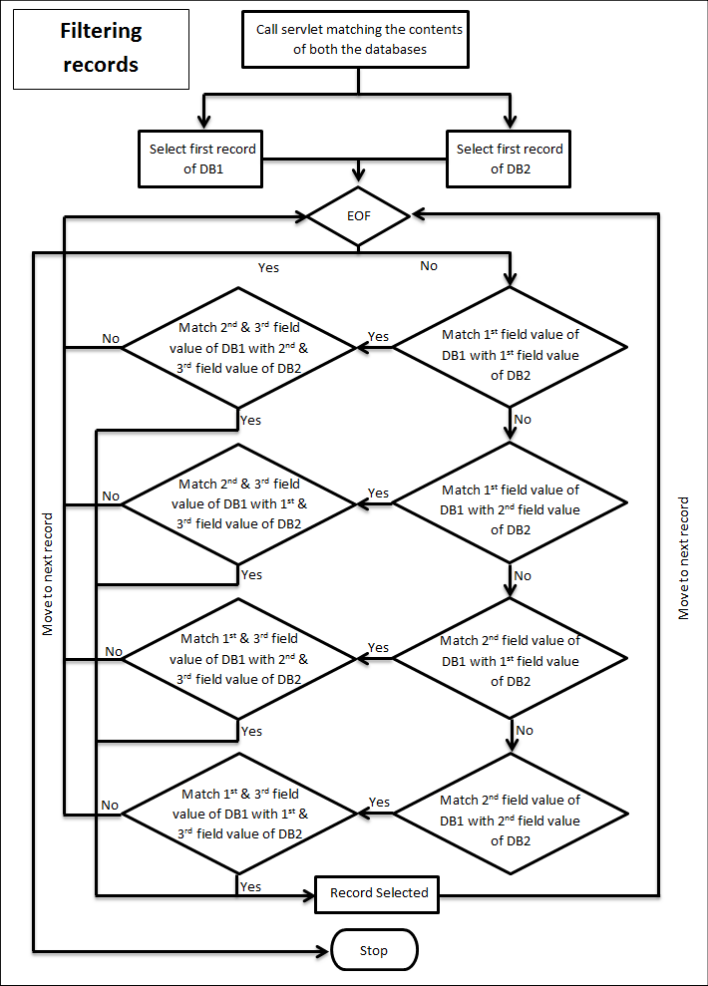
Flowchart describing algorithm of comparing both databases and filtering common records

**Figure 2C:**
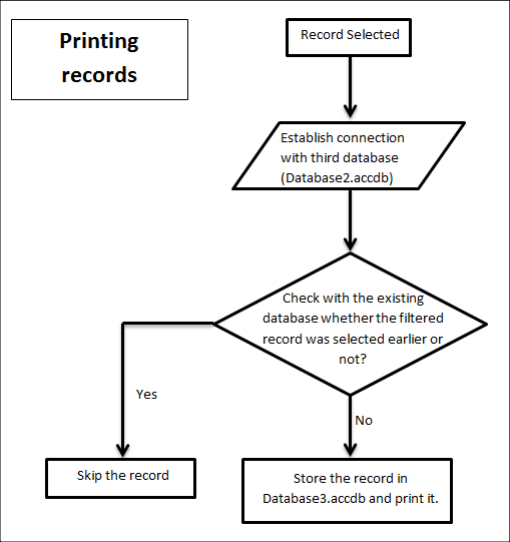
Flowchart describing algorithm for printing filtered records along with skipping duplicate filtration
